# Forty Years of the Ottawa Charter. Why Healthy Public Policy Still Matters for Health Equity in Australia

**DOI:** 10.1002/hpja.70216

**Published:** 2026-10-06

**Authors:** Carmel Williams, Samantha Thomas, Luke Wolfendon, Don Nutbeam

**Affiliations:** ^1^ Editor in Chief, Health Promotion Journal of Australia; ^2^ Centre for Health in All Policies Research Translation School of Public Health Adelaide University and South Australian Health and Medical Research Institute Adelaide Australia; ^3^ College of Medicine and Public Health Flinders University Adelaide Australia; ^4^ University of Canberra Canberra Australia; ^5^ Editor in Chief, Health Promotion International; ^6^ Public Health Institute for Health Transformation, Deakin University Melbourne Australia; ^7^ VicHealth Centre for the Commercial and Economics Determinants of Health Melbourne Australia; ^8^ Editor in Chief, Australian and New Zealand Journal of Public Health; ^9^ University of Newcastle Newcastle Australia; ^10^ Health Systems and Public Health Thematic Group Melbourne Australia; ^11^ National Centre of Implementation Science (NCOIS) Sydney Australia; ^12^ Sax Institute Sydney Australia; ^13^ Editor in Chief, Public Health Research Practice; ^14^ Public Health Sydney School of Public Health Sydney Australia

**Keywords:** health equity, health promotion, healthy public policy, Ottawa Charter, public health

## 1 Introuction

In November 2026, it will be 40 years since the first World Health Organization (WHO) International Conference on Health Promotion was held in Ottawa, Canada [1]. Its significance was captured in the development of the Ottawa Charter for Health Promotion [2], which has served as the guiding framework for the evolution of health promotion in the 40 years since publication [3]. This Editorial reflects on the enduring relevance of the Ottawa Charter's concept of healthy public policy for advancing health equity. We highlight Australia's important historical and ongoing contribution to this agenda and recognise the important role of public health research in generating the evidence, ideas and advocacy needed to drive policy change.

## 2 From Ottawa to Adelaide

Since the second half of the 19th century [1, 2, 3], public health legislation and regulation have been among the most powerful tools for protecting population health [4, 5]. Initially focused on sanitation and infectious disease control, public health policy evolved throughout the 20th century to confront the broader social, economic and environmental conditions that shape health. In doing so, governments recognised that health was not only produced within healthcare systems, but through the contexts in which people are born, live, work and age. This shift cemented public policy as a central tool for protecting population health and reducing health inequalities [6].

The decision to hold the Ottawa Conference in Canada reflected Canada's global leadership in public health evidence and action, driven largely by the influential ‘Lalonde Report’ [7] which directly challenged dominant biomedical models of health. The Lalonde Report marked a major political shift in understandings of health. It argued that healthcare services had limited impact on overall population health compared with other determinants and highlighted that increasing medical spending alone would not significantly improve health outcomes. These ideas were extended and refined by Nancy Milio and other critical public health scholars in the lead up to the Ottawa conference. Milio reframed health and health equity as a political and structural issue, arguing that policies related to income, employment, food systems, housing and education were fundamental drivers of these outcomes [8]. Milio is widely credited with popularising the use of the term ‘healthy public policy’ [8] to emphasise that improving health required coordinated approaches to health promotion across all areas and levels of government—concepts that helped to shape the intellectual foundations of the Ottawa Charter.

At the conclusion of the Ottawa conference, the Australian Government agreed to co‐host a second conference focused specifically on ‘Building Healthy Public Policy’ [9]. Aligned with the principles of the Charter, the work of Australia's Better Health Commission (1973–1976) had challenged the dominance of treatment‐focused healthcare and primed the Australian political system for greater investment in prevention, health promotion and system reform [10]. Held in Adelaide in 1988, this second conference sought to operationalise the Ottawa Charter's vision for healthy public policy [11]. The resulting Adelaide Statement emphasised that all areas of government should be accountable for the health and equity impacts of their decisions, and argued that creating healthy societies required coordinated action across all sectors—not just within health departments [12]. In doing so, the Adelaide conference helped to lay the foundations for approaches that remain highly influential today, including health impact assessments and the concept of Health in All Policies [12].

## 3 The Role of Political Action in Building Successful Healthy Public Policies

Australia has much to celebrate in its contribution to healthy public policy and is internationally recognised for pursuing legislative and regulatory reforms that have improved population health and reduced preventable harms. Most notably, is Australia's global leadership in tobacco control, introducing comprehensive legislative reforms that restricted tobacco advertising, sales and smoking in public places, alongside taxation policies designed to reduce consumption [13, 14]. In Victoria tobacco tax reforms also enabled the establishment of the world's first independent health promotion foundation, VicHealth which embedded prevention and health promotion within the policy architecture of government [15, 16]. Australia later became the first country in the world to introduce plain packaging legislation for tobacco products despite sustained opposition and legal threats from the tobacco industry [17] and more recently implemented some of the world strictest controls on vaping and other nicotine delivery products [18]. Importantly this willingness to act through public policy has extended beyond tobacco control. Areas of public health action that once seemed impossible were made possible through strong public health evidence, and political will—including HIV/AIDS prevention [19]; comprehensive immunisation systems (including HPV vaccinations which have eliminated cervical cancer) [20]; public health campaigns to prevent skin cancer [21]; gun control [22]; mandatory folic acid fortification [23] and most recently attempts to protect young people from the harms associate with social media platforms [24].

These policy successes did not emerge by chance. They were supported by considerable contributions to public health research, evidence and knowledge by Australian scientists and public health research and enabled by political leaders willing to confront powerful commercial, ideological and vested interests in the pursuit of population health goals. Australian Health Ministers such as Neil Blewett, Brian Howe and Nicola Roxon pursued ambitious and often politically contested reforms [25, 26, 27]. Prime Minister John Howard (1996–2007) demonstrated similar political resolve in implementing major gun control reforms following the Port Arthur massacre [22]. Evidence demonstrating the benefits of these and other world‐leading legislative and public health policy actions has seen them adopted in other jurisdictions globally. Effective policy action also depended on strong public institutions capable of developing, implementing and sustaining reform. Australia's relatively strong and stable public institutions provided the institutional and bureaucratic capacity needed to translate political ambition into legislation, regulation and implementation [28]. Equally important was the role of the health promotion and public health profession in identifying policy priorities, generating evidence and advocating for changes that improved health and well‐being outcomes [29]. Together, public health research, political leadership, capable public institutions and a strong health promotion workforce created the conditions for Australia's most significant healthy public policy achievements.

## 4 Mixed Success With Broader Whole of Government Ambitions

Whilst Australia has achieved significant success in responding to specific public health challenges, its record in implementing the broader vision of healthy public policy articulated in the Ottawa Charter has been far more uneven. Rather than isolated policy reforms, the Charter called for a whole of government commitment to health and equity—and agenda that later evolved into the Health in All Policies concept. This pattern has been evident across decades of reform efforts. From the Better Health Commission [30], to the National Health Goals and Targets [31] and Better Cities program [32] and the National Preventive Health Strategy [33] governments have repeatedly endorsed cross‐government responses to priority health issues. Yet these reforms have often lost momentum following political transition and shifting government priorities. They have also struggled to ensure effective implementation with the fidelity and research to ensure population level improvements in health. These experiences highlight a persistent challenge for healthy public policy. Whilst governments often endorse prevention rhetorically, sustaining long‐term structural reform requires political commitment that extends beyond electoral cycles and more comprehensive and effective implementation.

At a state level, some more consistent progress can be observed, most notably with implementation of the South Australian Health in All Policies Initiative, and development of the health lens analysis process [34, 35]. The South Australian HiAP initiative was designed to cast a health lens across government policies and priorities and seek opportunities for ‘win win’ policy outcomes that benefited both population heath and supported the achievement of the government's wider sectoral priorities [36, 37]. South Australian HiAP is regarded as one of the two leading examples globally on how to implement multisectoral action, and the WHO Health in All Policies 4 Pillars Model drew upon the lessons from the South Australian experience [38].

## 5 A Work in Progress

Whilst Australia has much to celebrate in its leadership on healthy public policy, progress has been uneven, politically contested and often fragile. One less widely reported moment of the Adelaide Conference was the disruption of the opening ceremony by First Nations advocates, protesting the profound inequities experienced by Aboriginal and Torres Strait Islander peoples [11]. As Prime Minister Bob Hawke took the stage to officially open the event, activists exposed a central contradiction—a nation recognised for progressive public health leadership, whilst Indigenous communities continued to experience drastically lower life expectancies, poor living conditions and systemic racism [39]. Despite decades of concerted whole of government efforts to ‘Close the Gap’ in the health, social and economic outcomes for Indigenous Australians, these inequities persist [40, 41]. This remains a stark reminder of the need for sustained structural reform where Indigenous ways of ‘knowing, being and doing can be safely applied across an array of contexts, for the benefit of all’ [42].

Similarly, Australia's public health successes have been arguably highly selective. There has been very little political resolve to tackle areas such as alcohol regulation [43], gambling reform [44, 45] and the climate crisis and planetary health [46, 47]. In these areas, governments have repeatedly retreated in the face of powerful industry lobbying, political influence and the revolving door between politics, government and corporate interests [48]. The result has been policy inertia precisely where stronger regulation has been most needed to protect health and reduce inequities. The challenge of ‘lifestyle drift’ remains one of the greatest challenges for health promotion [49]. Governments routinely acknowledge the social and structural determinants of health, yet priorities and funding are often directed to education campaigns and behaviour change programmes. The focus on behaviour and lifestyle is attractive as it centres responsibility with the individual—and not with government or the private sector. There is a real risk that the benefits of digital technology to provide interactive, personalised support and advice will further exacerbate ‘lifestyle drift’ and divert attention from equally important structural determinants of health require societal responses in the form of public policy [50].

At the same time, the systems needed to sustain healthy public policy are being weakened. Prevention and health promotion systems globally are consistently underfunded, with public health efforts often receiving only a fraction of the investment directed towards biomedical and clinical research or tertiary healthcare services [51, 52]. This chronic underinvestment in public health threatens the independent evidence base needed to inform, defend and advocate for healthy public policy, and is associated with a range of methodological and cultural challenges in the local political, social and commercial contexts. For example, the flow of information and finances is increasingly impacted by commercial actors at all levels influencing the public narrative, which, in turn, affects political and investment priorities. The underinvestment of public health and health promotion research reflects broader political decisions about which forms of knowledge, expertise and innovation governments choose to value, and weakens the capacity of governments and communities to respond to emerging threats to health equity.

## 6 Conclusion

Australia has demonstrated leadership in healthy public policy over the past 40 years. From this experience we have learned that impactful policy reform requires sustained political commitment, strong public institutions, properly funded prevention and research systems and a health promotion community prepared to continue advocating for structural change. As Editors in Chief, we believe public health and health promotion journals are part of the political and social infrastructure needed to defend prevention, challenge inequity and hold governments and commercial actors accountable for health harms. Healthy public policy has always been political. Progress over the next 40 years will depend on whether governments are willing to act accordingly.

## Funding

The authors have nothing to report.

## Conflicts of Interest

The authors declare no conflicts of interest.

## Data Availability

Data sharing not applicable to this article as no datasets were generated or analysed during the current study.

## References

[1] World Health Organization , “Health Determinants, Promotion and Prevention,” 2026, https://www.who.int/teams/health‐promotion/enhanced‐wellbeing/first‐global‐conference.

[2] World Health Organization , “Ottawa Charter for Health Promotion,” Health Promotion International 1, no. 4 (1986): 405.

[3] S. L. Thomas , I. Kickbusch , M. Kökény , and O. Okan , “40 Years of the Ottawa Charter for Health Promotion—Reaffirming Health for All,” Health Promotion International 40, no. 4 (2025): daaf143. 10.1093/heapro/daaf143 40836494

[4] M. Heenan , M. Ralph , and J. Shanthosh , “Why Public Health Acts Could Be Our Best Chance to Stem the Tide of Non‐Communicable Diseases,” Australian and New Zealand Journal of Public Health 48, no. 6 (2024): 100208. 10.1016/j.anzjph.2024.100208 39616690

[5] R. A. Goodman , R. E. Hoffman , W. Lopez , G. W. Matthews , M. A. Rothstein , and K. L. Foster , Law in Public Health Practice (Oxford University Press, 2006).

[6] G. Carey and B. Crammond , “Action on the Social Determinants of Health: Views From Inside the Policy Process,” Social Science and Medicine 128 (2015): 134–141. 10.1016/j.socscimed.2015.01.024 25616195

[7] M. Lalonde , A New Perspective on the Health of Canadians: A Working Document (Minister of National Health and Welfare, 1974).

[8] N. Milio , “Making Healthy Public Policy; Developing the Science by Learning the Art: An Ecological Framework for Policy Studies,” Health Promotion International 2, no. 3 (1987): 263–274. 10.1093/heapro/2.3.263 10303001

[9] Department of the Prime Minister and Cabinet , ed., “Speech by the Prime Minister Conference on “Healthy Public Policy” Adelaide—5 April 1988,” in Department of the Prime Minister and Cabinet (Adelaide, 1988).

[10] R. F. Southby , “Health Care Reform: Looking Back to Go Ahead,” Medical Journal of Australia 189, no. 1 (2008): 33–34. 10.5694/j.1326-5377.2008.tb01892.x 18601639

[11] B. Lunnay , F. Baum , C. Angelo , and G. Crawford , “Continuing the Vision: Engaging the Ottawa Charter for Future ‘Healthy Public Policy’,” Health Promotion International 40, no. 5 (2025). 10.1093/heapro/daaf178 PMC12530321 41100676

[12] Health Promotion International , “The Adelaide Recommendations: Healthy Public Policy,” Health Promotion 3, no. 2 (1988): 183–186.

[13] Department of Health, Disability and Ageing , “Tobacco Control: Australian Government,”.

[14] World Health Organization , WHO Report on the Global Tobacco Epidemic, 2025 (WHO, 2025).

[15] VicHealth , “Our History,” 2026, https://www.vichealth.vic.gov.au/about‐us/our‐history.

[16] R. Galbally , Australia: A Firm Foundation for Tobacco Control: The Victorian Health Promotion Foundation Model (Springer, 2000).

[17] M. Daube , A. R. Moodie , and S. Chapman , “Plain Packaging of Tobacco Products—Plainly a Success,” Medical Journal of Australia 197, no. 10 (2012): 537–538. 10.5694/mja12.11612 23163666

[18] B. Freeman , “10 Years of Preventive Health in Australia. Part 4—Extending Gains in Tobacco Control,” Public Health Research and Practice 35, no. 2 (2025): PU24019. 10.1071/PU24019 40456590

[19] T. Kirby , “Australia Marks 40 Years Since Its First Diagnoses of HIV,” Lancet HIV 9, no. 7 (2022): e460–e461. 10.1016/S2352-3018(22)00170-9 35777408

[20] S. Yuill , C. Naidu , M. Smith , et al., “Cervical Cancer Elimination in Australia and the Asia Pacific: Progress and Barriers,” Medical Journal of Australia 224, no. 4 (2026): e70164. 10.5694/mja2.70164 PMC13036706 41914308

[21] H. Walker , C. Maitland , T. Tabbakh , P. Preston , M. Wakefield , and C. Sinclair , “Forty Years of Slip! Slop! Slap! A Call to Action on Skin Cancer Prevention for Australia,” Public Health Research and Practice 32, no. 1 (2022): e31452117. 10.17061/phrp31452117 35290993

[22] S. Chapman , P. Alpers , K. Agho , and M. Jones , “Australia's 1996 Gun Law Reforms: Faster Falls in Firearm Deaths, Firearm Suicides, and a Decade Without Mass Shootings,” Injury Prevention 12, no. 6 (2006): 365–372. 10.1136/ip.2006.013714 PMC2704353 17170183

[23] H. D'Antoine and C. Bower , “Folate Status and Neural Tube Defects in Aboriginal Australians: The Success of Mandatory Fortification in Reducing a Health Disparity,” Current Developments in Nutrition 3, no. 8 (2019): nzz071. 10.1093/cdn/nzz071 PMC6642066 31346585

[24] J. Fardouly , “Potential Effects of the Social Media Age Ban in Australia for Children Younger Than 16 Years,” Lancet Digital Health 7, no. 4 (2025): e235–e236. 10.1016/j.landig.2025.01.016 40148007

[25] B. Howe , “Reflections on Making Public Policy (1977–1996),” Australian Journal of Social Issues 59, no. 4 (2024): 905–921.

[26] N. Blewett , “Valuing the Past … Investing in the Future,” Australian and New Zealand Journal of Public Health 20, no. 4 (1996): 343–345. 10.1111/j.1467-842x.1996.tb01042.x 8908753

[27] T. Kirby , “Nicola Roxon: Australia's Minister for Health and Ageing,” Lancet 373, no. 9669 (2009): 1073. 10.1016/S0140-6736(09)60637-1 19328990

[28] K. Dowding and A. Martin , eds., “Political Institutions and Policy in Australia,” in Policy Agendas in Australia (Springer International Publishing, 2017), 59–83.

[29] R. M. Caron , K. Noel , R. N. Reed , J. Sibel , and H. J. Smith , “Health Promotion, Health Protection, and Disease Prevention: Challenges and Opportunities in a Dynamic Landscape,” AJPM Focus 3, no. 1 (2024): 100167. 10.1016/j.focus.2023.100167 PMC10749873 38149078

[30] Better Health Commission , Looking Forward to Better Health (Australian Govt. Pub. Service, 1986).

[31] D. Nutbeam , M. Wise , A. Bauman , E. Harris , and S. Leeder , Goals and Targets for Australia's Health in the Year 2000 and Beyond: Report Prepared for the Commonwealth Department of Health, Housing & Community Services, Rev. ed. (Public Health Dept., University of Sydney, 1993).

[32] L. Neilson , “The ‘Building Better Cities’ Program 1991–96: A Nation‐Building Initiative of the Commonwealth Government,” 2008, https://press‐files.anu.edu/downloads/press/p118181/pdf/ch089.pdf.

[33] Department of Health, Disability and Ageing , National Preventive Health Strategy 2021–2030 (Commonwealth of Australia, 2021).

[34] F. Baum , T. Delany‐Crowe , C. MacDougall , et al., “To What Extent Can the Activities of the South Australian Health in All Policies Initiative Be Linked to Population Health Outcomes Using a Program Theory‐Based Evaluation?,” BMC Public Health 19, no. 1 (2019): 88. 10.1186/s12889-019-6408-y PMC6339362 30658616

[35] A. P. Lawless , C. Williams , C. Hurley , D. Wildgoose , A. Sawford , and I. Kickbusch , “Health in All Policies: Evaluating the South Australian Approach to Intersectoral Action for Health,” Canadian Journal of Public Health 103 (2012): eS15–eS19. 10.1007/BF03404454 PMC6973620 23618043

[36] H. van Eyk , E. Harris , F. Baum , T. Delany‐Crowe , A. Lawless , and C. MacDougall , “Health in All Policies in South Australia‐Did It Promote and Enact an Equity Perspective?,” International Journal of Environmental Research and Public Health 14, no. 11 (2017): 1288. 10.3390/ijerph14111288 PMC5707927 29068400

[37] I. Kickbusch and K. Buckett , Implementing Health in All Policies: Adelaide 2010 (Government of South Australia, 2010).

[38] World Health Organization , Working Together for Equity and Healthier Populations: Sustainable Multisectoral Collaboration Based on Health in All Policies Approaches (World Health Orgnaization, 2023), Report No.: ISBN 978‐92‐4‐006753‐0 (electronic version) ISBN 978‐92‐4‐006754‐7 (print version).

[39] B. S. Hetzel , “Historical Perspectives on Indigenous Health in Australia,” Asia Pacific Journal of Clinical Nutrition 9, no. 3 (2000): 157–163. 10.1046/j.1440-6047.2000.00139.x 24394449

[40] C. J. Bond and D. Singh , “More Than a Refresh Required for Closing the Gap of Indigenous Health Inequality,” Medical Journal of Australia 212, no. 5 (2020): 198–9.e1. 10.5694/mja2.50498 32030749

[41] R. Maddox , C. Kornacki , S. K. Bradbrook , and T. Calma , “The Ottawa Charter: Indigenous Sovereignty, Resistance, and Health Promotion at 40,” Health Promotion International 40, no. 6 (2025): daaf195. 10.1093/heapro/daaf195 PMC12641176 41277447

[42] P. Saunders , P. Dudgeon , M. Kennedy , K. M. Kong , J. T. Hughes , and O. Pearson , “Indigenous Health Special Issue 2025: Carving Our Path With Spirit, Strength and Solidarity,” Medical Journal of Australia 223, no. 1 (2025): 28–29. 10.5694/mja2.52693 40621684

[43] M. McInerney , Australian Politicians Urged to Find the Political Courage to Reform Alcohol and Drug Laws (Croakey Health Media, 2022).

[44] S. Thomas , H. Pitt , S. McCarthy , M. Murray , and M. Daube , “Australia's Proposed Gambling Reforms May Be Good for Industry, but Not for Public Health,” Health Promotion International 41, no. 2 (2026): daag057. 10.1093/heapro/daag057 PMC13107171 42028844

[45] S. L. Thomas , G. Crawford , M. Daube , et al., “Time for Policies on Gambling to Benefit Health—Not the Gambling Industry,” Health Promotion Journal of Australia 34, no. 2 (2023): 267–271. 10.1002/hpja.721 37038275

[46] R. Xu , P. Yu , Y. Liu , et al., “Climate Change, Environmental Extremes, and Human Health in Australia: Challenges, Adaptation Strategies, and Policy Gaps,” Lancet Regional Health—Western Pacific 40 (2023): 100936. 10.1016/j.lanwpc.2023.100936 PMC10730315 38116505

[47] J. Nalau , “Australia Has No Plan for Climate Change Adaptation. Why? The Diplomat,” 2024, https://thediplomat.com/2024/01/australia‐has‐no‐plan‐for‐climate‐change‐adaptation‐why/.

[48] S. Thomas , M. Daube , M. van Schalkwyk , et al., “Acting on the Commercial Determinants of Health,” Health Promotion International 39, no. 6 (2024): daae183. 10.1093/heapro/daae183 PMC11579602 39569487

[49] M. Robinson and J. A. Smith , “The Lazy Language of ‘Lifestyles’,” Health Promotion Journal of Australia 34, no. 1 (2023): 3–5. 10.1002/hpja.677 36424767

[50] D. Nutbeam , “Artificial Intelligence and Health Literacy—Proceed With Caution,” Health Literacy and Communication Open 1, no. 1 (2023): 2263355.

[51] B. N. Latif , L. Coombe , T. Driscoll , A. van Zwieten , C. Sherrington , and S. Khalatbari‐Soltani , “Public Health and Prevention Research Within the Medical Research Future Fund,” Australian and New Zealand Journal of Public Health 48, no. 4 (2024): 100171. 10.1016/j.anzjph.2024.100171 39085012

[52] A. Shiell , K. Garvey , S. Kavanagh , V. Loblay , and P. Hawe , “How Do We Fund Public Health in Australia? How Should We?,” Australian and New Zealand Journal of Public Health 48, no. 5 (2024): 100187. 10.1016/j.anzjph.2024.100187 39306556

